# Multidisciplinary Consensus Prostate Contours on Magnetic Resonance Imaging: Educational Atlas and Reference Standard for Artificial Intelligence Benchmarking

**DOI:** 10.1016/j.ijrobp.2025.03.024

**Published:** 2025-03-26

**Authors:** Yuze Song, Anna M. Dornisch, Robert T. Dess, Daniel J.A. Margolis, Eric P. Weinberg, Tristan Barrett, Mariel Cornell, Richard E. Fan, Mukesh Harisinghani, Sophia C. Kamran, Jeong Hoon Lee, Cynthia Xinran Li, Michael A. Liss, Mirabela Rusu, Jason Santos, Geoffrey A. Sonn, Igor Vidic, Sean A. Woolen, Anders M. Dale, Tyler M. Seibert

**Affiliations:** a Department of Radiation Medicine and Applied Sciences, University of California San Diego, La Jolla, California;; b Department of Electrical and Computer Engineering, University of California San Diego, La Jolla, California;; c Department of Radiation Oncology, University of Michigan, Ann Arbor, Michigan;; d Department of Radiology, Cornell University, Ithaca, New York;; e Department of Clinical Imaging Sciences, University of Rochester Medical Center, Rochester, New York;; f Department of Radiology, University of Cambridge, Cambridge, United Kingdom;; g Radformation, New York, New York;; h Department of Urology, Stanford School of Medicine, Palo Alto, California;; i Department of Radiology, Massachusetts General Hospital, Boston, Massachusetts;; j Department of Radiation Oncology, Massachusetts General Hospital, Boston, Massachusetts;; k Department of Radiology, Stanford School of Medicine, Palo Alto, California;; l Institute for Computational and Mathematical Engineering, Stanford University, Palo Alto, California;; m Department of Urology, University of Texas Health Sciences Center San Antonio, San Antonio, Texas;; n Department of Biomedical Data Science, Stanford University, Palo Alto, California;; o Quibim, New York, New York;; p Cortechs.ai, San Diego, California;; q Department of Radiology and Biomedical Imaging, University of California San Francisco, San Francisco, California;; r Department of Radiology, University of California San Diego, La Jolla, California;; s Department of Neurosciences, University of California San Diego, La Jolla, California;; t Halιcιoğlu Data Science Institute, University of California San Diego, La Jolla, California;; u Department of Bioengineering, University of California San Diego, La Jolla, California;; v Department of Urology, University of California San Diego, La Jolla, California

## Abstract

**Purpose:**

Evaluation of artificial intelligence (AI) algorithms for prostate segmentation is challenging because ground truth is lacking. We aimed to: (1) create a reference standard data set with precise prostate contours by expert consensus, and (2) evaluate various AI tools against this standard.

**Methods and Materials:**

We obtained prostate magnetic resonance imaging cases from six institutions from the Qualitative Prostate Imaging Consortium. A panel of 4 experts (2 genitourinary radiologists and 2 prostate radiation oncologists) meticulously developed consensus prostate segmentations on axial T_2_-weighted series. We evaluated the performance of 6 AI tools (3 commercially available and 3 academic) using Dice scores, distance from reference contour, and volume error.

**Results:**

The panel achieved consensus prostate segmentation on each slice of all 68 patient cases included in the reference data set. We present 2 patient examples to serve as contouring guides. Depending on the AI tool, median Dice scores (across patients) ranged from 0.80 to 0.94 for whole prostate segmentation. For a typical (median) patient, AI tools had a mean error over the prostate surface ranging from 1.3 to 2.4 mm. They maximally deviated 3.0 to 9.4 mm outside the prostate and 3.0 to 8.5 mm inside the prostate for a typical patient. Error in prostate volume measurement for a typical patient ranged from 4.3% to 31.4%.

**Conclusions:**

We established an expert consensus benchmark for prostate segmentation. The best-performing AI tools have typical accuracy greater than that reported for radiation oncologists using computed tomography scans (the most common clinical approach for radiation therapy planning). Physician review remains essential to detect occasional major errors.

## Introduction

Artificial intelligence (AI) tools are being developed and deployed rapidly, especially for medical imaging.^1^ AI tools are valuable additions to clinical medicine in an era of increasingly vast data.^2^ However, adverse consequences may follow if AI tools are deployed without proper stewardship by the medical community. Expert medical professionals must set the bar to judge the performance of AI tools their field will use.

One area of increasing interest is the development of AI algorithms for prostate auto-segmentation, an important task for diagnostic radiologists, radiation oncologists, and urologists. Establishing acceptable accuracy and precision metrics requires knowledge about what constitutes a clinically meaningful error. In diagnostic radiology, the most common segmentation applications are the measurement of prostate volume for Prostate Specific Antigen (PSA) density,^3,4^ whole gland segmentation for fusion biopsy, and target segmentation for fusion biopsy. The PSA density task does not require as much accuracy as more difficult applications (measuring extraprostatic extension risk). Although many prostate auto-segmentation tools for magnetic resonance imaging (MRI) are available, most were designed and validated to assist with segmentation for MRI-ultrasound fusion biopsy.^5^ It is unknown how well any available auto-segmentation tool works against a true gold standard, because there is no consensus guideline for prostate delineation on MRI. This has particular implications in radiation therapy, where small errors can impact patient outcomes (a randomized trial showed differences in prostate contours as small as 2 mm can measurably increase toxicity).^6^ An improved standard is needed for the accuracy of prostate delineation for radiation therapy planning and can also serve as the reference for the evaluation of prostate auto-segmentation tools.

Excellent reference standard contours are a prerequisite for a meaningful comparison and properly validated tool. Our objectives are 2-fold: (1) create a reference standard data set with highly accurate prostate contours via expert consensus and (2) meticulously evaluate an array of AI tools for prostate segmentation on MRI against the defined consensus reference standard. In accomplishing the first objective, we present a detailed contouring guide for physician education.

## Methods and Materials

### Cases

We obtained patient cases from six institutions from the Quantitative Prostate Imaging Consortium (QPIC) as part of a study of prostate cancer detection approved by the institutional review board at each institution.^7,8^ All patients underwent prostate multiparametric MRI for known or suspected prostate cancer. We included at least 10 cases from each institution. Cases in the database were selected from each institution in sequential order with 2 modifications. First, we excluded cases with prominent artifacts (hip implants, prominent bowel gas, and/or very large body habitus that interfered with image quality). Second, the data set was enriched to ensure approximately one-third of cases from each site had a prominent median lobe (prostate extension into the bladder) because this is common in clinical practice, and it is important to understand how automated tools perform for common anatomic variations. Thus, once the desired number of “all-comer” cases was reached for each site, we specifically included additional cases with a large median lobe to ensure adequate representation of this common anatomical variant in the data set.

### Reference standard contour development

We convened a panel of 4 experts to develop the reference standard prostate segmentation data set. Our panel included 2 genitourinary (GU) radiologists (with 12 and 21 years of experience, each surpassing the number of MRIs reported to qualify as prostate MRI experts^9^) and 2 GU radiation oncologists specializing in MRI-guided prostate radiation therapy (10 years of experience each). One of the GU radiation oncologists, also a prostate MRI researcher, generated initial contours on high-resolution axial T_2_-weighted slices. The panel met weekly from March to June 2024 to review the initial contours slice-by-slice. All 3 planes were always visualized, and high-resolution coronal and sagittal T_2_-weighted acquisitions were available. If any panel member judged the contour deviated >1 mm from the true prostate boundary, the panel deliberated until consensus. Each slice case was reviewed at least twice. All members of the panel in attendance agreed on the final contour for each case. All 4 panelists were generally present for case reviews; meetings only proceeded when at least 3 panelists were present.

### Prostate contouring ground rules

Before the consensus review, the panel decided on ground rules for situations with more than one reasonable approach. If partial volume effects were present but the prostate was clearly visible, this was included as the prostate. The proximal seminal vesicles (SVs) were not intentionally included. If a visible plane divided the SVs from the prostate, the tissue was labeled as SV and excluded. Tissue with SV appearance but without a clear dividing plane on an axial slice was included as the prostate. Although this may lead to the inclusion of a small proportion of SVs, a rule was needed, and overestimation was preferable to undercovering the prostate. Additionally, the panel agreed this tissue would be indistinguishable from the prostate on computed tomography (CT) and was invariably included in prior trials of prostate cancer radiation therapy using CT for treatment planning. The tip of the apex may not be perfectly visualized on MRI because axial slices are recommended to be 3.0-mm thick (in-plane resolution should be 0.7 × 0.4 mm).^10^ Therefore, at the apex, any suspicion of visible prostate, even with only subtle partial volume effects, was included as the prostate.

### Auto-segmentation models

We invited 8 companies with commercially available prostate auto-segmentation tools to participate and 3 companies (company A, company B, and company C) accepted. Each commercially available tool has FDA clearance and/or CE marking for prostate auto-segmentation. Additionally, we evaluated 3 deep-learning models developed at academic centers (UCSD and Stanford). Stanford Model 1 employs a vision transformer backbone pretrained using the vision foundation model DINOv2,^11^ coupled with a segmentation head. This architecture was refined through the incorporation of patch-level contrastive learning techniques (Stanford Model 2).^12^ Development and validation of the UCSD model is described below. None of the tools were trained or previously validated using any cases in our curated reference standard data set.

### UCSD model development

We collected 618 cases from 5 different data sets for training, including 92 cases from the multisite data set,^13^ 52 cases from NCI-ISBI,^14^ 139 cases from Prostate158,^15^ 197 cases from Reimagine,^16^ 45 cases from Cortechs.ai customer data (courtesy of Cortechs.ai), 30 cases from Prostate-3T challenge,^17^ 50 cases from PROMISE12 challenge,^18^ and 13 cases from the Cancer Imaging Archive (PROSTATE-DIAGNOSIS, 9 cases^19^ and Fused Radiology-Pathology Prostate, 4 cases^20^). The training process used nnU-Net,^21^ a robust and well-established model architecture. We implemented a 5-fold cross-validation strategy, with a total of 1000 training epochs. The initial learning rate was 0.01 and gradually decreased to 0.00002 by the final epoch. Training was conducted on a single NVIDIA GeForce RTX 2080 Ti GPU, with a batch size of 2 per GPU. The input T_2_-weighted volumes were cropped to a patch size of 16 × 320 × 320 and resampled to a uniform voxel size of 3.0 × 0.4 × 0.4 mm^3^.

### Evaluation of AI tool performance

We compared each AI model segmentation to the expert consensus prostate contour of the reference standard data set. We compared segmentation overlap using the mean Dice score of the entire prostate, with 1 indicating perfect overlap and 0 indicating no overlap. We used a variety of clinically relevant metrics. To measure how accurately AI tools defined the prostate’s superior extent, we compared the difference in MRI slice number containing the superior-most contour between each model versus the reference standard. We repeated this to evaluate how well AI tools defined the prostate’s inferior extent. Additionally, we measured how far the AI tool strayed outside the prostate (max error outside [mm]) and cut into the prostate (max error inside [mm]). We calculated the margin required to encompass the entire prostate’s average error by comparing each auto-segmentation to a generated distance map based on the boundary of the reference standard. We determined the absolute and relative volume differences between each auto-segmentation and the reference standard, reported in mL and percentage, respectively. Because uncertainty is greatest at the superior-most and inferior-most slices, we defined the “main gland” as everything but the top 2 and bottom 2 slices of the reference standard prostate. We compared each tool’s accuracy over the main gland by measuring Dice scores. We also conducted a qualitative review of the clinical acceptability of the results of the best-performing model via the consensus opinion of a 3-person panel of GU radiation oncologists, each with 10 years of experience (further details are available in Appendix E1). Because the purpose of this analysis is educational and not to market or disparage any commercial product, we will not label the commercial results with company names.

## Results

### Cases

We included prostate contours for 68 cases for the reference standard data set. Cases came from 6 imaging centers, and MRI data were acquired using 3 distinct 3T scanners by 2 vendors (GE Healthcare; Siemens Healthineers) (Table 1, Table E2). None of the cases used an endorectal coil. Cases were initially screened (subjectively) by one of the investigators and excluded if there were obvious and severe artifacts. There were 3 such exclusions. All had artifacts from significant rectal gas changes during interleaved slice acquisition, resulting in very disjointed 3D volumes. All cases that passed initial screening were reviewed by the full panel in detail and were deemed to have clinically usable quality by all panel members.

**Fig. 1.**
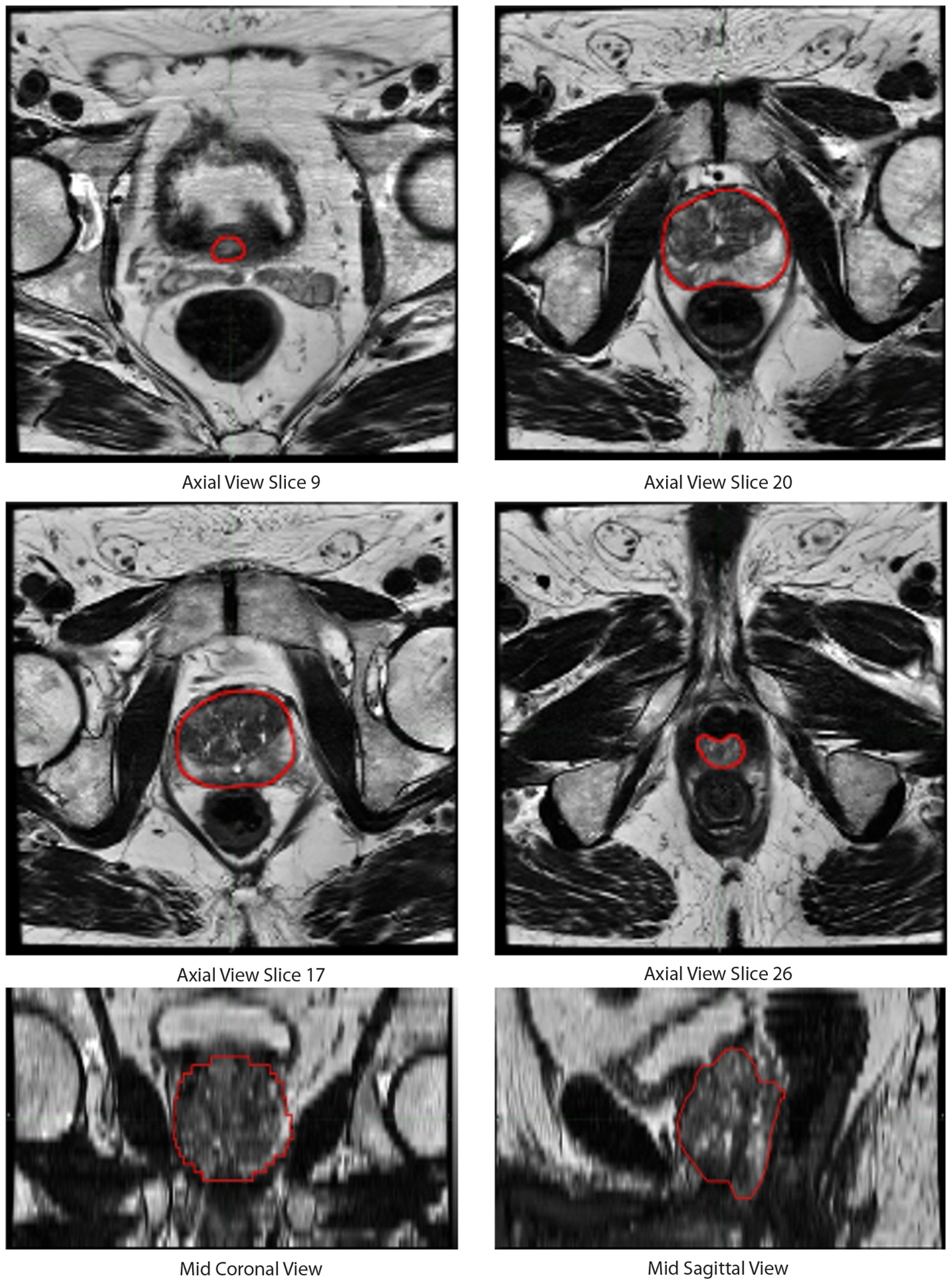
Expert-defined consensus prostate contour on magnetic resonance imaging for a representative patient case. The expert contour is shown in red contour on axial T_2_-weighted slices.

**Fig. 2.**
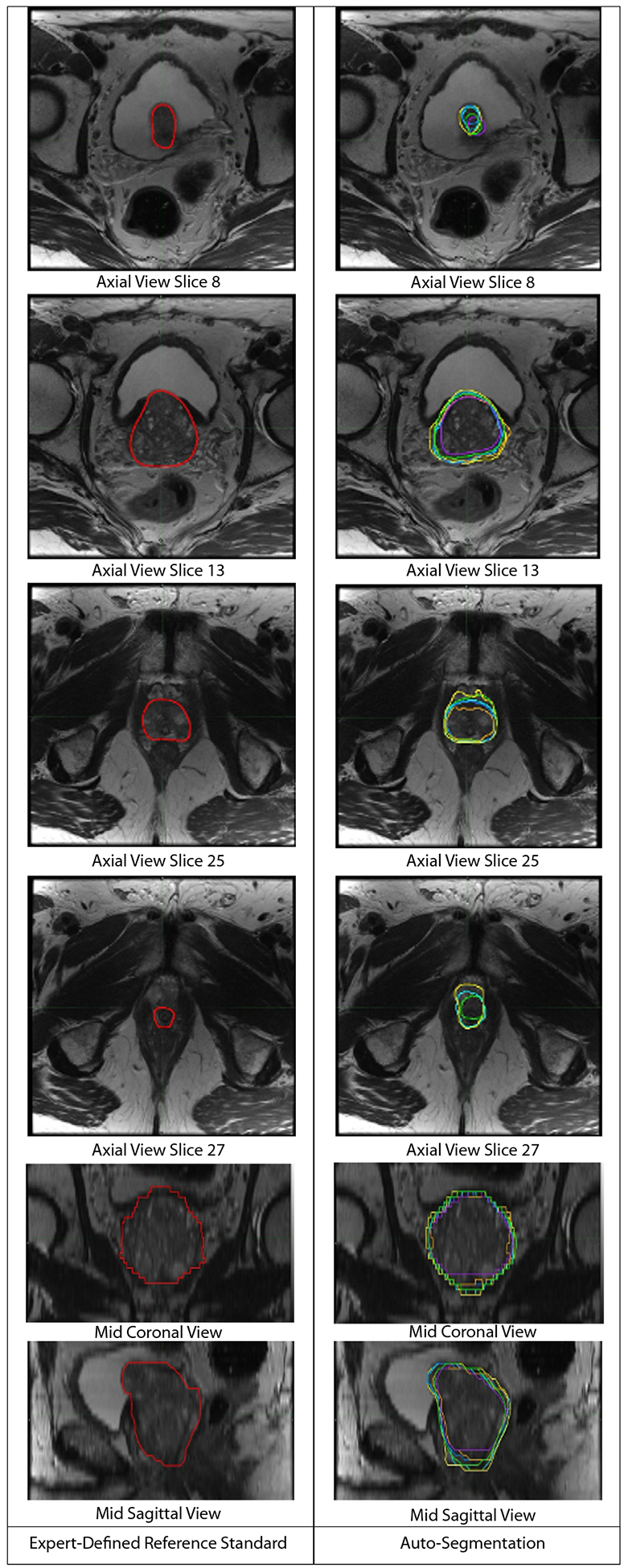
Visual comparison of expert-defined consensus contour versus all auto-segmentation models for axial T_2_-weighted slices from a single patient case. The left panel represents the expert-defined consensus prostate contour, shown in red contour. The right panel shows the corresponding auto-segmentation products for all models for each slice. Blue: UCSD; yellow: Stanford Model 1; cyan: Stanford Model 2; green: company A’s product; orange: company B’s product; and purple: company C’s product.

The panel reached a consensus on prostate contours on each slice of all 68 cases. We present all slices for 2 patients to illustrate complete prostate volumes and serve as contouring guides (representative slices, Figs. 1 and 2). These are available for download as slide decks in PowerPoint and as DICOM files.

### Qualitative observations

The greatest uncertainty regarding prostate boundary was in determining the inferior-most slice containing the prostate. There was also some uncertainty at the base, although this was generally clearer than the apex. Occasionally, distinguishing the dorsal venous plexus from the prostate was dif-ficult, especially when the plexus extended posteriorly. The panel was satisfied with the final consensus contours but recognized there was some uncertainty, and these cases sometimes required more time to be confident the prostate boundary was accurate. Lastly, we sometimes debated what was neurovascular bundle versus prostate when determining the posterolateral prostate boundary. On one hand, the most common cause of uncertainty in any part of the prostate segmentation was partial volume effects from 3.0 mm slices. On the other hand, the partial volume effects between slices amount to interpolation between slices and were generally judged to have only a small impact on the overall prostate contour shape. On qualitative review of the contours of the best-performing model, 75% were found to be clinically acceptable without any modification. The remaining 25% of cases were judged to need modification (often at the apex) to be clinically acceptable, although the expert panel agreed that the necessary modifications were typically limited and that the errors often likely fall within the range of contouring variability expected among practicing radiation oncologists. Additional qualitative results can be found in Table E1.

### Evaluation of AI performance

All 6 models correctly identified the general location of the prostate in all 68 reference standard cases, except for one commercial model, which failed to generate auto-segmentations for 3 of 68 (4.4%) cases. Those 3 cases were from 2 different imaging centers. All models had greater variation at the apex and base compared to midgland. AI auto-segmentations are illustrated for one patient in Figure 2. For the whole prostate, the median (across patients) Dice score for the 6 AI models ranged from 0.80 (0.72–0.85) to 0.94 (0.92–0.95) (Table 2). All 6 models were more accurate in the main gland with median Dice-main ranging from 0.83 (0.75–0.87) to 0.95 (0.95–0.96) (Table 2).

At the prostate’s superior boundary, the auto-segmentation models differed from the reference standard by a range of 1 (0–1) to 1 (1–2) slices (median [IQR]) for the best- and worst-performing models, respectively. At the inferior boundary of the prostate, the auto-segmentation models differed from the reference standard by a range of 1 (0–1) to 3 (2–4) slices, highlighting greater error in defining the inferior extent.

The best-performing model typically included at least one point 3.0 mm outside the true prostate for each patient (compared with 9.4 mm for the worst-performing model). The median (IQR across cases) maximum error outside the prostate ranged from 3.0 mm (2.0–4.0) for the best-performing model to 9.4 mm (6.7–12.5) for the worst-performing model.

The best-performing model typically excludes 3.0 mm of true prostate at least one point somewhere in the prostate. The median (IQR) maximum error inside the prostate ranged from 3.0 mm (2.8–4.5) for the best-performing model to 8.5 mm (6.8–9.3) for the worst-performing model.

At any given point in the prostate, the expected error in either direction (inside or outside the prostate) was 1.3 mm for a typical patient for the best-performing model and 2.4 mm for a typical patient for the worst-performing model. The median (IQR) mean error ranged from 1.3 mm (1.2–1.5) to 2.4 mm (2.1–2.8).

**Fig. 3.**
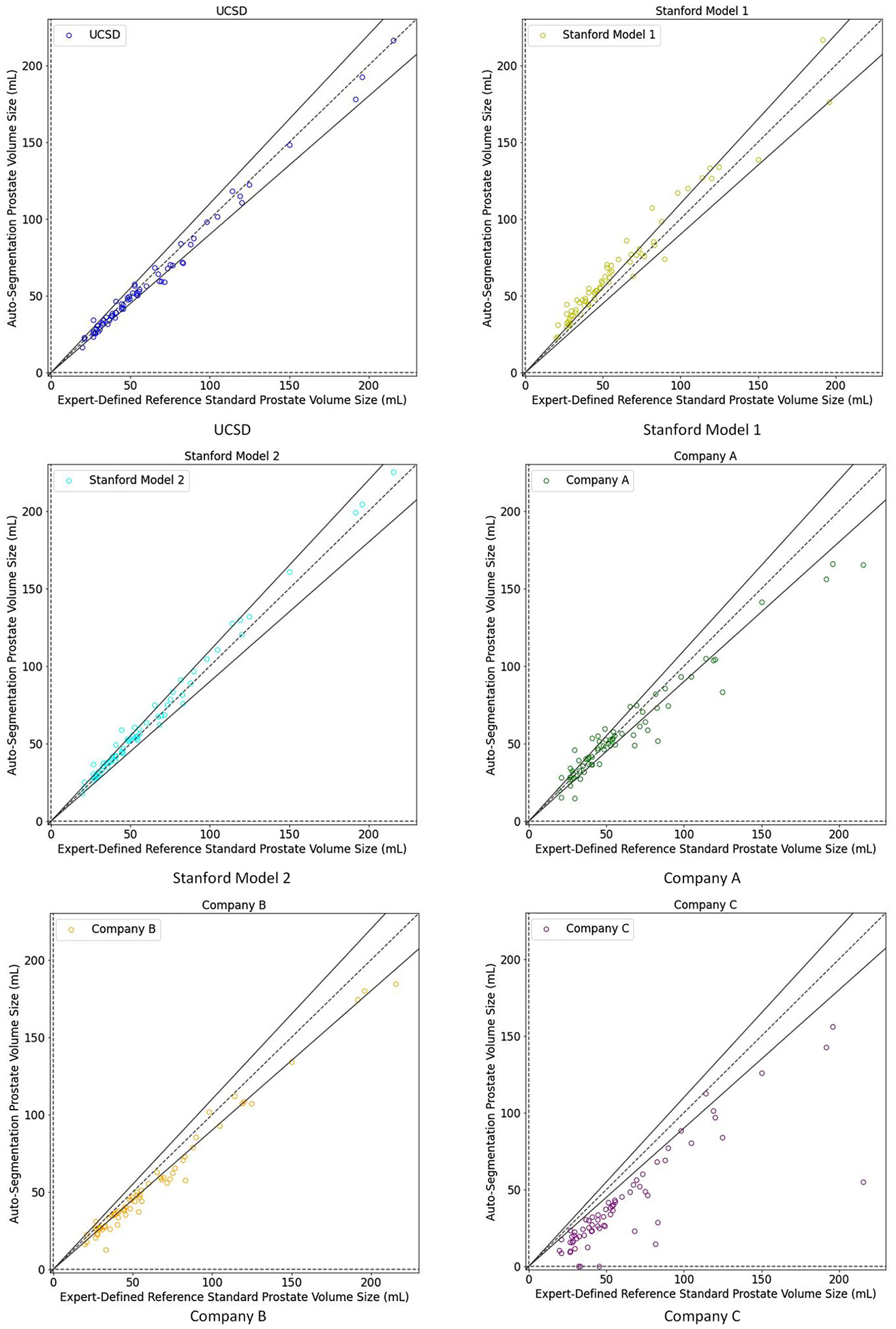
Scatter plot of auto-segmentation volume versus expert-defined consensus contour. N = 68 cases. For all panels, the X-axis shows the absolute prostate volume (mL) of the expert-defined consensus contour whereas the Y-axis shows the absolute prostate volume (mL) of the auto-segmentation product. If the result falls between the 2 solid lines, the relative volume of the auto-segmentation is within 10% (in either direction) of the expert-defined volume. Blue: UCSD; yellow: Stanford Model 1; cyan: Stanford Model 2; green: company A’s product; orange: company B’s product; and purple: company C’s product.

The median (IQR) relative volume difference across patients ranged from 4.3% (2.3%−7.7%) for the best-performing model to 31.4% (22.2%−43.8%) for the worst-performing model (Table 2). The best-performing model typically gives estimates of prostate volume within 2.3% to 7.7% of the true value. The worst-performing model typically gives estimates that differ by 22% to 44% from the true value. Most models underestimated prostate volume (Fig. 3).

The presence of a prominent median lobe or radiographic T3a carcinoma did not appear to be a major driver of inaccuracy for the models (Figs. E1 and E2 and Tables E3, E4, E5, and E6).

**Fig. 4.**
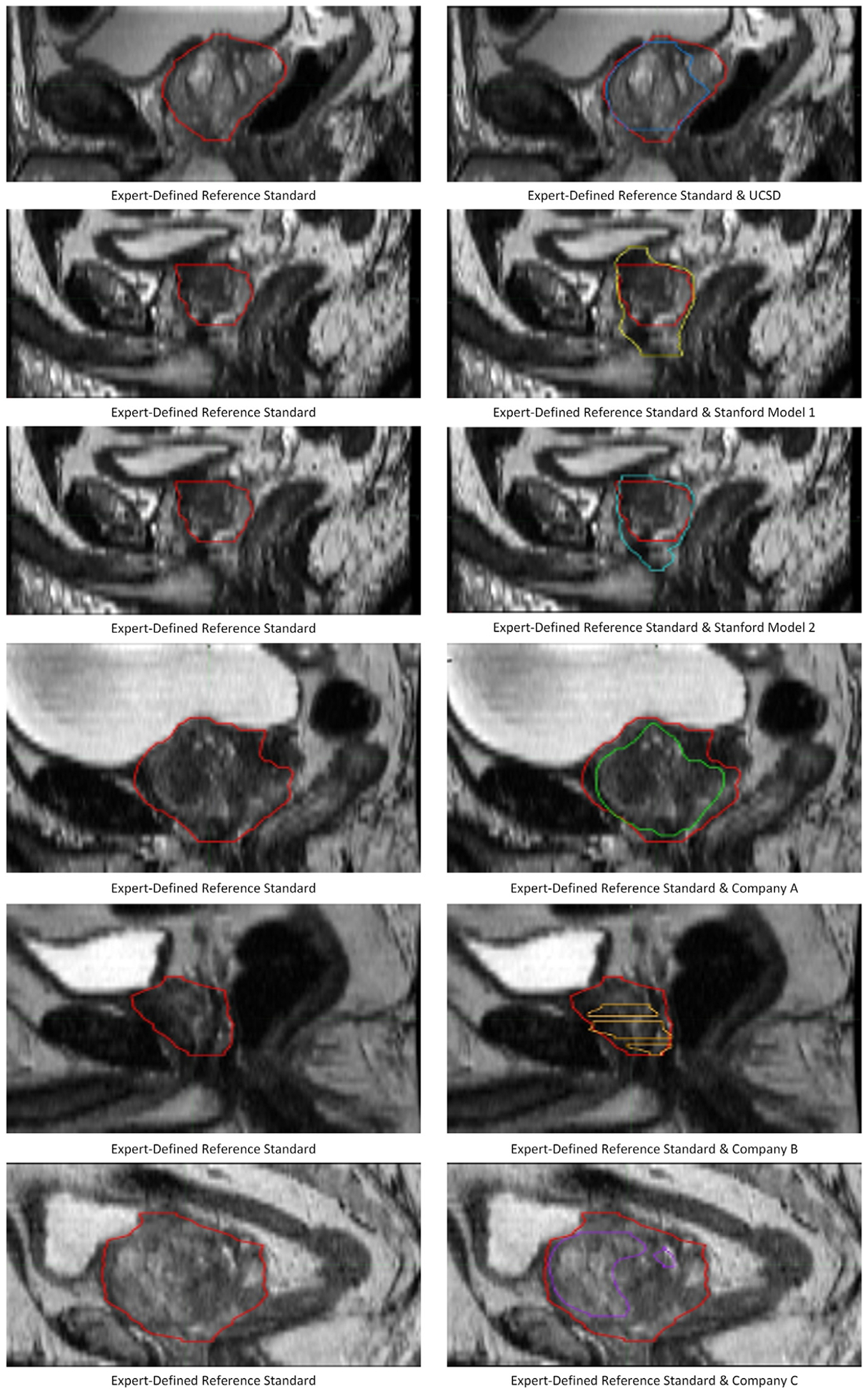
Example auto-segmentation errors. Example slices illustrating particularly bad auto-segmentation errors from the cases with the worst Dice score for each artificial intelligence tool’s prostate segmentation. These are single slices from cases shown in the sagittal view. In all panels, the red contour is the expert-defined consensus contour. Blue: UCSD; yellow: Stanford Model 1; cyan: Stanford Model 2; green: company A’s product; orange: company B’s product; and purple: company C’s product.

Lastly, although a model’s overall performance may be quite good, there can still be considerable variation across cases. We provide representative images showing the case with the lowest Dice score for each of the auto-segmentation models (Fig. 4).

## Discussion

Amidst a flood of published and available AI tools, the medical community needs to define the reference standard against which AI models are judged. Here, we created a reference standard data set of 68 cases through consensus determination by an interdisciplinary expert panel for rigorous evaluation of prostate auto-segmentation AI tools. We present 2 cases in full as educational resources for a wide audience (radiology, radiation oncology, urology, etc.).

Using the expert consensus data set, we tested 6 AI tools (3 commercially available and 3 academic). All 6 models provided useful results. Prostate volume was typically adequate for calculating PSA density. However, even the best-performing models had a volume error of >10% for some patients, suggesting that currently, these tools require physician supervision. None of the AI tools universally gave results accurate enough for radiation therapy without manual review and revision if errors >2.0 mm are considered important. The randomized MIRAGE trial compared CT-guided stereotactic body radiation therapy with a 4.0 mm planning margin to MRI-guided stereotactic body radiation therapy with a 2.0 mm planning margin for treatment of localized prostate cancers. Participants treated with the larger margin had significantly worse GU and gastrointestinal toxicity, suggesting errors in prostate contours as small as 2.0 mm could affect outcomes.^6^

Physician contouring of the prostate shows substantial variation, and errors are common.^22,23^ For radiation therapy planning, radiation oncologists have traditionally performed prostate segmentation on CT images. One study found radiation oncologists’ prostate contours were, on average, 30% larger than the true prostate volume, although still only including 84% of the prostate.^24^ Another study reviewed the manual contours of 300 prostates and described numerous and varied errors in each part of the prostate.^23^ Here, one of the best-performing AI tools had a median Dice score of 0.94, and the mean error over the full prostate contour was typically only 1.3 mm, likely clinically acceptable for radiation therapy planning and more accurate than the reported accuracy of physicians contouring the prostate on CT images.^25^

Forming our reference standard was time-consuming and required considerable investment by experts. We reinforce the importance of multidisciplinary collaboration in developing reference standards for meaningful comparisons of AI tools. Here, the GU radiologists were experienced at reviewing high numbers of prostate MRIs and were well-trained in cross-sectional anatomy. The radiation oncologists provided the clinical context of prostate contours used for radiation therapy. Radiation oncologists strive for highly accurate contours over the full prostate in their routine clinical work, whereas approximate volumes may be acceptable for radiologists’ reporting volumes for deriving PSA density. The radiation oncologists contributed insights around SVs in the context of prostate cancer treatment, the inclusion of the median lobe, and concerns about missing the tip of the apex with the use of 3.0 mm MRI slices. Importantly, multidisciplinary collaboration created a better reference standard than either group would have accomplished alone. All panelists were emphatic that the development of the reference standard data set was a highly educational exercise.

According to a review of 100 commercially available AI products for medical imaging, only 36% had peer-reviewed evidence of efficacy.^26^ Validating AI tools is significantly challenging but necessary to ensure that patients and health care professionals can trust their accuracy.^27^ We maintain that there is great value in a reference standard data set independent of any data used for training the AI tools it is meant to test.^23,28^ This avoids inflation of accuracy because of model overfitting. Reference standard data sets should be carefully curated and only used for validation of models developed elsewhere, permitting objective head-to-head performance evaluations of existing and future AI tools. Furthermore, different AI tool outputs require different statistical evaluation methods.^29^ Accuracy evaluation should include indices of overall agreement (Dice score^30^) and of clinically relevant errors (deviations from the reference standard inside and outside the prostate).

Our study has practical value for radiation oncologists and patients today. MRI-based radiation therapy planning has been shown to improve precision and may limit toxicity compared to commonly used CT-based approaches.^23,31^ Delineation of the prostate on MRI is also integral to focal radiation boost for prostate cancer, which reduces cancer recurrence and metastatic spread.^32,33^ However, many radiation oncologists are unfamiliar^34^ and/or not proficient^35,36^ at contouring the prostate on MRI. Both our MRI-based consensus contouring guides (Figs. 1 and 2) and validation of MRI-based auto-segmentation tools provide unique but related tools to facilitate widespread, accurate adoption of MRI-based radiation therapy planning.

Our study has some limitations. First, there is no purely objective way to verify the precise in vivo prostate boundary; hence, expert consensus is the best that can be achieved. Theoretically, a larger expert group could be engaged, but larger groups naturally complicate scheduling and dilute each member’s contribution. Second, acquisition protocols for prostate MRI vary across centers/scanners. We mitigated this by including data from 6 centers and 2 vendors with 3 different scanner models. We used diagnostic MRI data from Prostate Imaging-Reporting and Data System (PIRADS) compliant protocols rather than acquisitions designed for radiation therapy planning because this is the most common. Third, we excluded cases with poor image quality arising from hip implants, prominent bowel gas, and/or very large body habitus; thus, the performance of AI tools in these circumstances is unknown. Finally, although outside the scope of this study, clinical use of prostate segmentation for radiation therapy has additional considerations, including the following: (1) physiological changes to prostate position between or within treatments, and (2) the need for accurate registration of MRI to CT when a separate planning CT simulation is used.

## Conclusions

To our knowledge, we present the first expert consensus guide for prostate radiation therapy planning using MRI and a multi-institutional, interdisciplinary expert consensus data set for meticulous evaluation of auto-segmentation AI tools. We found that some currently available AI tools are generally highly accurate, achieving average errors of <2.0 mm. Physician review remains necessary, as all AI tools make clinically meaningful errors in some cases.

## Supplementary Material

supplementary material

Supplementary material associated with this article can be found in the online version at doi:10.1016/j.ijrobp.2025.03.024.

## Figures and Tables

**Table 1:** Characteristics of the cases included in this study

Case characteristics	Total study MRI images (n = 68)
Cohorts	
UC San Diego (UCSD)	10
Harvard University’s Massachusetts General Hospital (MGH)	14
University of Rochester Medical Center (URMC)	12
UC San Francisco (UCSF)	10
UT Health Sciences Center San Antonio (UTHSCSA)	10
University of Cambridge	12
Cases with prominent median lobe vs selected without regard to anatomy	
Median lobe	23
Sequential, without regard to anatomy	45

Institution	MRI Scanner models	No. of scanners
UCSD	GE Healthcare Discovery MRI750, GE Healthcare Signa Premier	3
URMC	SIEMENS Skyra	2
MGH	GE Healthcare Signa Premier	1
UCSF	GE Healthcare Signa Premier	2
Cambridge	GE Healthcare Discovery MRI750	1
UTHSCSA	SIEMENS Skyra	2
Total	3 Models	11 Scanners

*Abbreviations:* MRI = magnetic resonance imaging.

**Table 2:** Dice, Max error outside the prostate (ie, how far the AI segmentation strayed beyond the true prostate, in mm), Max error inside the prostate (ie, how far the AI segmentation cut into the true prostate, in mm), average error (mm), Dice-main, difference in the superior extent of contour (number of slices), difference in the inferior extent of contour (number of slices), and volume difference (%) of each model

Model	Dice			Max error outside the prostate (mm)			Max error inside the prostate (mm)			Average error (mm)		
	Median			Median			Median			Median		
Model	Min	IQR	Max	Min	IQR	Max	Min	IQR	Max	Min	IQR	Max
UCSD		0.94			3.0			4.0			1.3	
	0.87	0.92–0.95	0.97	1.3	3.0–3.9	7.8	0.5	3.0–6.0	10.3	0.9	1.2–1.5	2.4
Stanford Model 1		0.89			7.0			3.0			2.0	
	0.75	0.87–0.91	0.94	3.2	6.0–9.0	15.7	0.55	2.8–4.5	16.7	1.1	1.7–2.2	4.6
Stanford Model 2		0.92			9.4			3.4			2.1	
	0.64	0.90–0.93	0.95	3.0	6.7–12.5	30.0	1.6	3.0–4.7	11.0	1.1	1.6–2.6	8.3
Company A		0.90			4.4			4.5			1.7	
	0.65	0.87–0.91	0.94	1.7	3.2–5.8	15.8	1.6	5.8–6	11.0	1.1	1.5–2.0	3.3
Company B		0.89			5.0			6.4			1.9	
	0.34	0.86–0.91	0.93	2.7	3.7–6.5	21.3	3.7	5.9–8.3	14.9	1.3	1.7–2.2	3.9
Company C		0.80			3.0			8.5			2.4	
	0.00	0.72–0.85	0.91	0.0	2.0–4.0	41.4	0.0	6.8–9.3	24.2	0.0	2.1–2.8	30.7
Model	Dice-main			Difference in the superior extent of contour (slices)			Difference in the inferior extent of contour (slices)			Volume difference (%)		
	Median			Median			Median			median		
Model	Min	IQR	Max	Min	IQR	Max	Min	IQR	Max	Min	IQR	Max
UCSD		0.95			1			1			4.7	
	0.90	0.95–0.96	0.98	0	0–1	2	0	0–2	3	0.1	3.0–8.3	27.1
Stanford Model 1		0.92			0			1			16.4	
	0.81	0.90–0.93	0.96	0	0–1	3	0	1–2	5	4.9	10.2–22.9	64.7
Stanford Model 2		0.94			1			3			4.3	
	0.72	0.93–0.95	0.96	0	0–1	4	0	2–4	9	0.1	2.3–7.7	36.1
Company A		0.92			1			1			9.2	
	0.67	0.89–0.93	0.95	0	0–1	3	0	0–1	3	0.1	4.8–17.9	54.5
Company B		0.91			1			1			12.0	
	0.36	0.89–0.92	0.95	0	1–2	8	0	0–1	5	0.8	8.1–16.8	62.0
Company C		0.83			1			3			31.4	
	0.00	0.75–0.87	0.93	0	0–2	4	0	2–4	9	1.6	22.2–43.8	100.0

*Abbreviations:* AI = artificial intelligence; Max = maximum; Min = minimum; IQR = interquartile range.

The median, Min, IQR, and Max refer to across patients for that metric/model combination. For example, the UCSD model gave a Dice score of 0.97 for the patient where it was most accurate, 0.87 for the patient where it was least accurate, and 0.94 was the median Dice for the UCSD model across all 68 patients.

## Data Availability

Research data are stored in an institutional repository and will be shared on request to the corresponding author.
