# Supplementary material for: Multidisciplinary Consensus Prostate Contours on Magnetic Resonance Imaging: Educational Atlas and Reference Standard for Artificial Intelligence Benchmarking
Source: Int J Radiat Oncol Biol Phys. Author manuscript; Available in PMC 2025 Jul 10. (PMC12243632; doi:10.1016/j.ijrobp.2025.03.024)
Supplement: supplementary material [file NIHMS2092091-supplement-supplementary_material.docx]

**Supplementary Material**

**Qualitative Analysis (Appendix E1)**

Methods

We convened a panel of three experts to qualitatively evaluate the clinical acceptability of the prostate auto-segmentations of the best performing model (UCSD). Our panel included three GU radiation oncologists specializing in MRI-guided prostate radiotherapy (10 years’ experience each). The panel reviewed the auto segmentation of the UCSD model compared to the expert consensus prostate contour for each case of the reference standard dataset on high-resolution axial *T_2_*-weighted slices. All three planes were always visualized. The panel rated each auto-segmentation on a scale of 1 to 3 with the following definitions: (1) clinically unacceptable; (2) clinically acceptable and equivalent to what can be achieved contouring on CT; (3) clinically acceptable with excellent contour on MRI. For clinically unacceptable cases (score of 1) a modifier of apex only was given to cases which otherwise would have received a higher score but due to inferior performance at only the apex were judged to be unacceptable.

Results

| Score | Cases, N (%) |
| --- | --- |
| Clinically Acceptable (Total) | 51 (75%) |
| 3 | 28 (41%) |
| 2 | 23 (34%) |
| Clinically Unacceptable (Total) | 17 (25%) |
| 1 | 6 (9%) |
| 1 (apex only) | 11 (16%) |

Table E1. Quantitative Analysis of Clinical Acceptability of the UCSD Model Auto-segmentations. Each auto-segmentation rated on a scale of 1 to 3. A score of 1 is clinically unacceptable and scores of 2 and 3 are clinically acceptable with 2 indicating performance equivalent to contouring on CT and 3 indicating excellent performance on MRI.

| **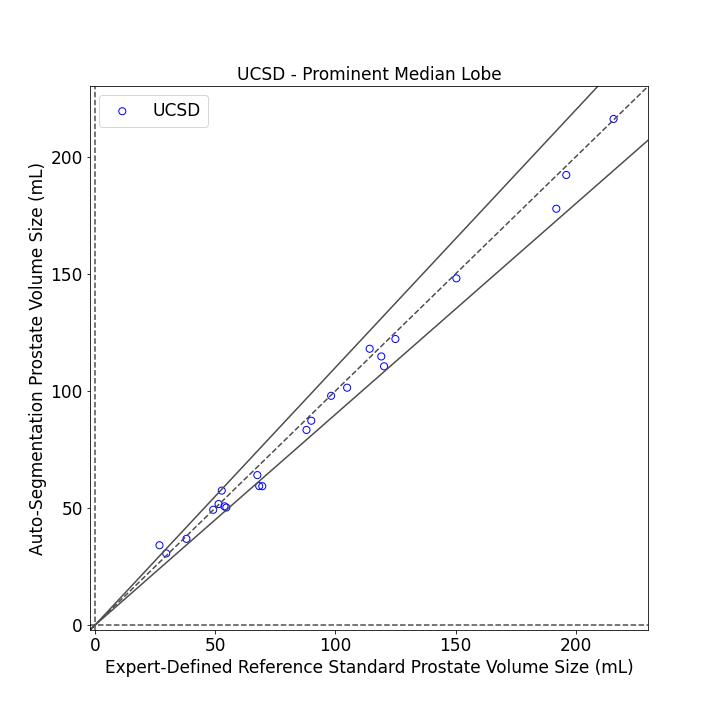**  A  B  C  D  UCSD | **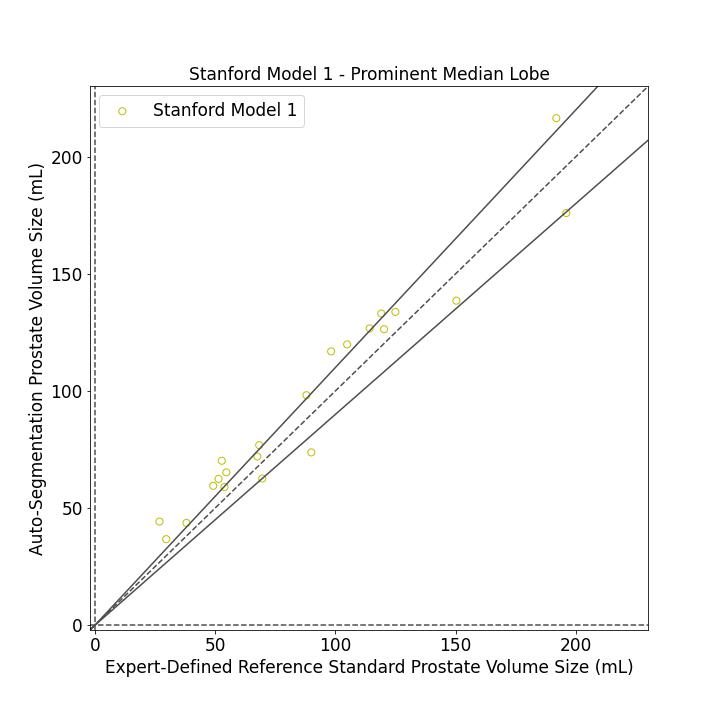**  Stanford Model 1 |
| --- | --- |
| **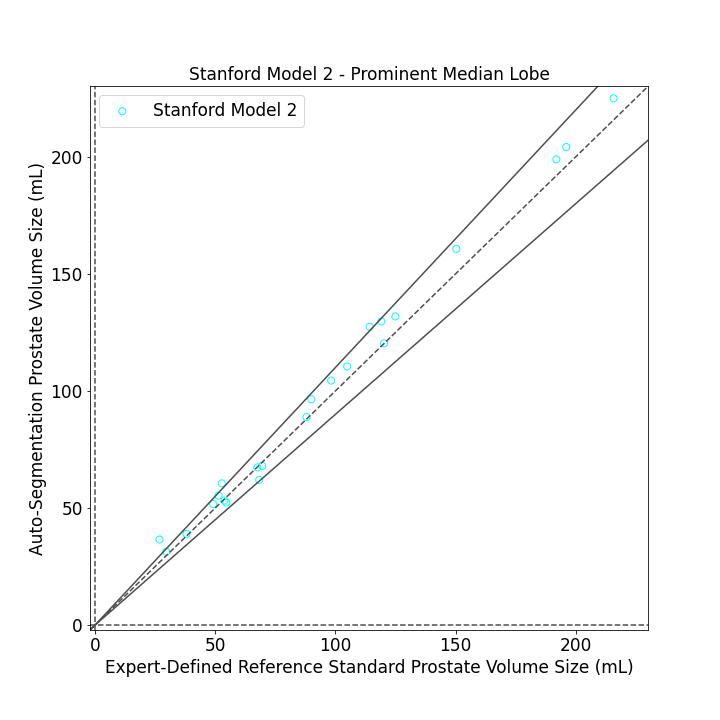**  Stanford Model 2 | **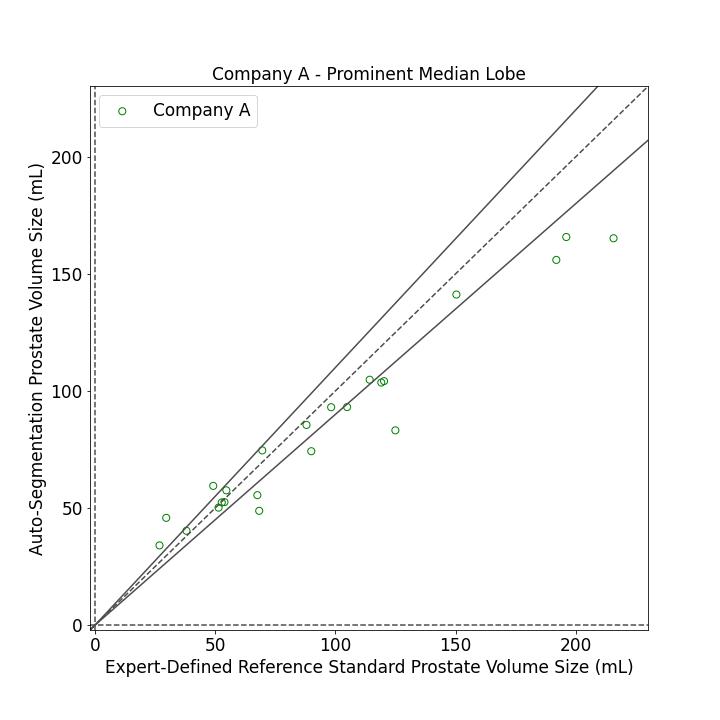**  Company A |
| **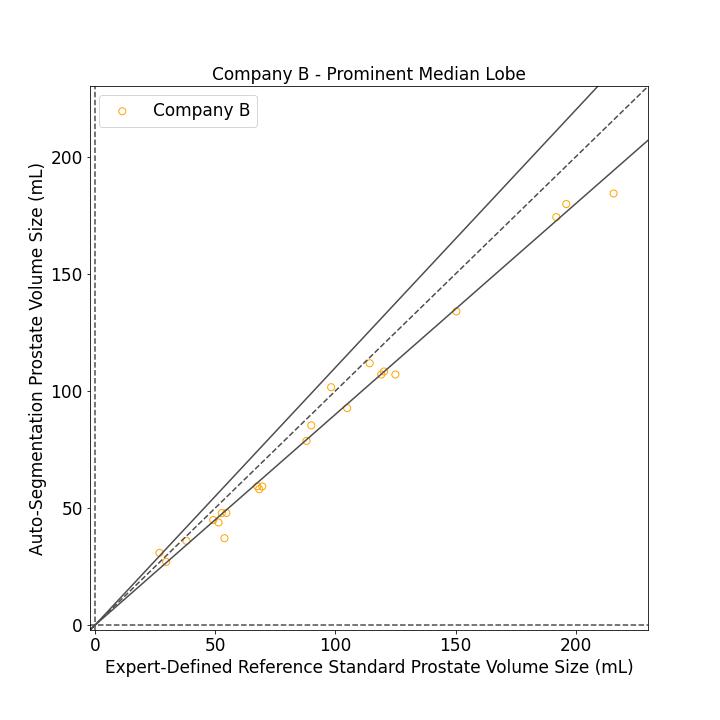**  E  Company B | **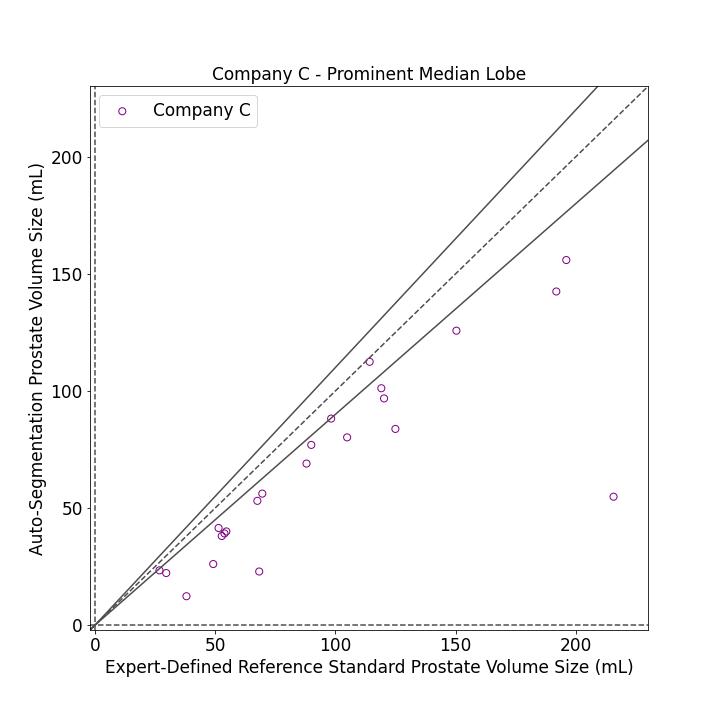**  F  Company C |
| **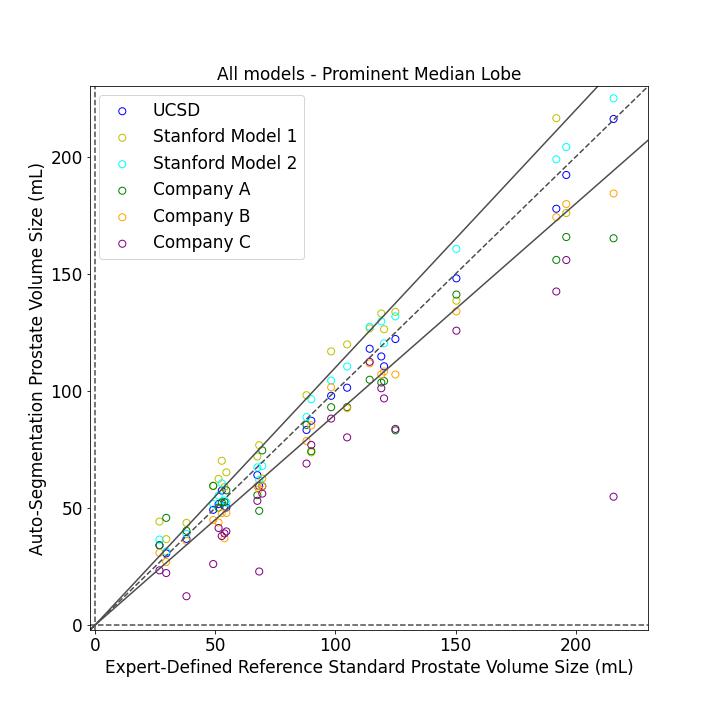**  G  All Models | |

Figure E1.

Scatter plot of auto-segmentation volume vs. expert-defined consensus contour for cases with a prominent median lobe. N=23 cases.

For all panels, the X-axis shows the absolute prostate volume (mL) of the expert-defined consensus contour while the Y-axis shows the absolute prostate volume (mL) of the auto-segmentation product for the cases specifically selected for their median lobe. Panel G compares all auto-segmentation models versus the consensus contour while panels A-F compare each model individually against the expert contour. Blue: UCSD. Yellow: Stanford Model 1. Cyan: Stanford Model 2. Green: Company A’s product. Orange: Company B’s product. Purple: Company C’s product.

| **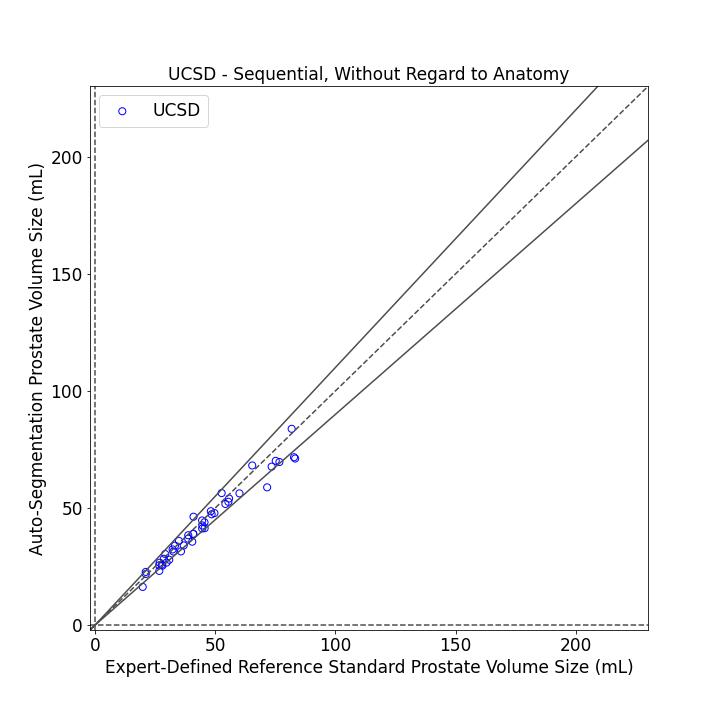**  A  B  C  D  UCSD | **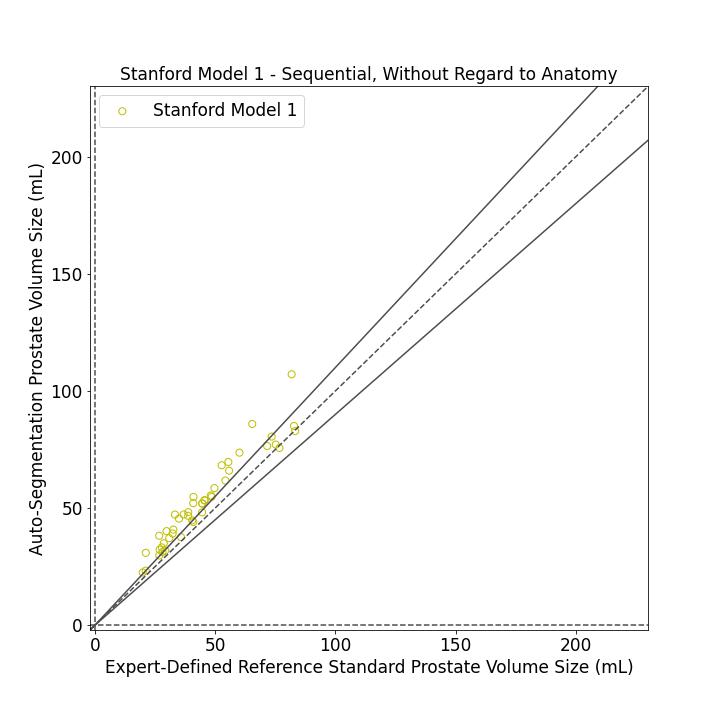**  Stanford Model 1 |
| --- | --- |
| **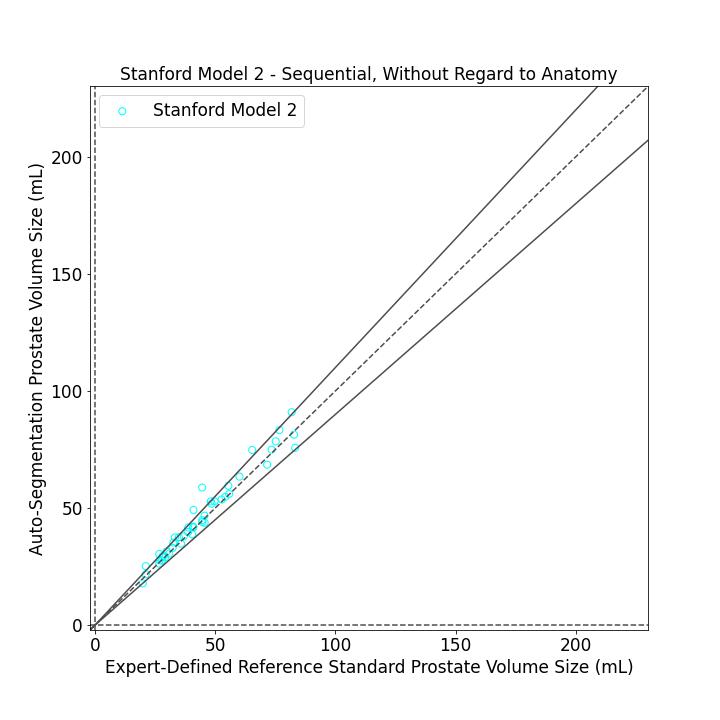**  Stanford Model 2 | **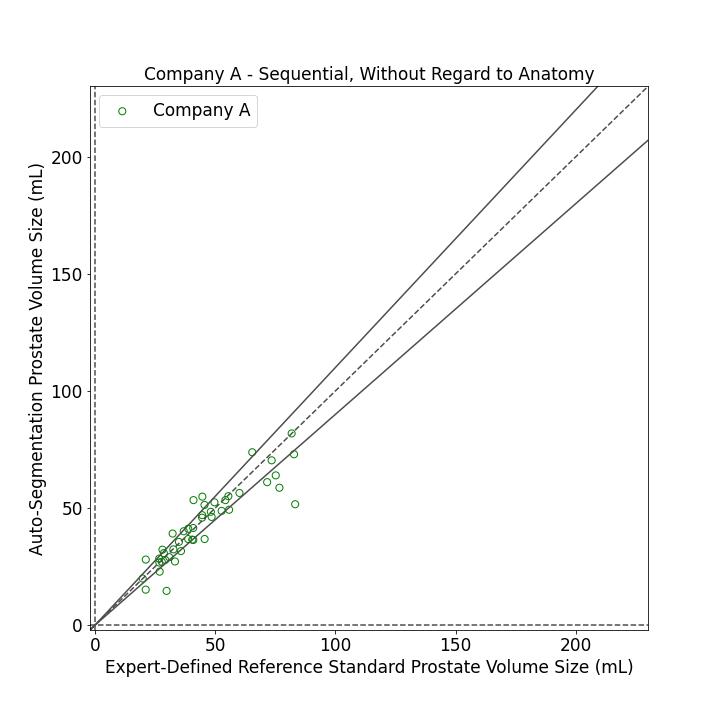**  Company A |
| **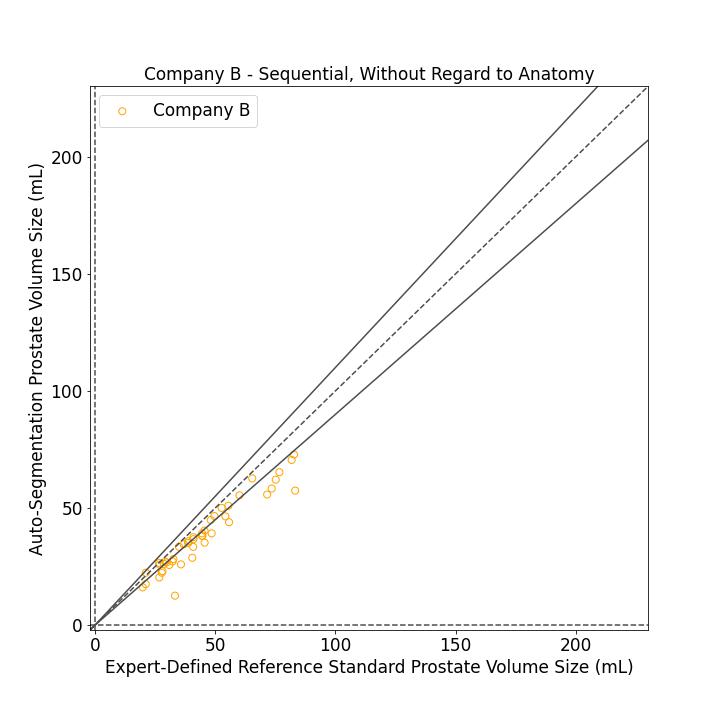**  E  Company B | **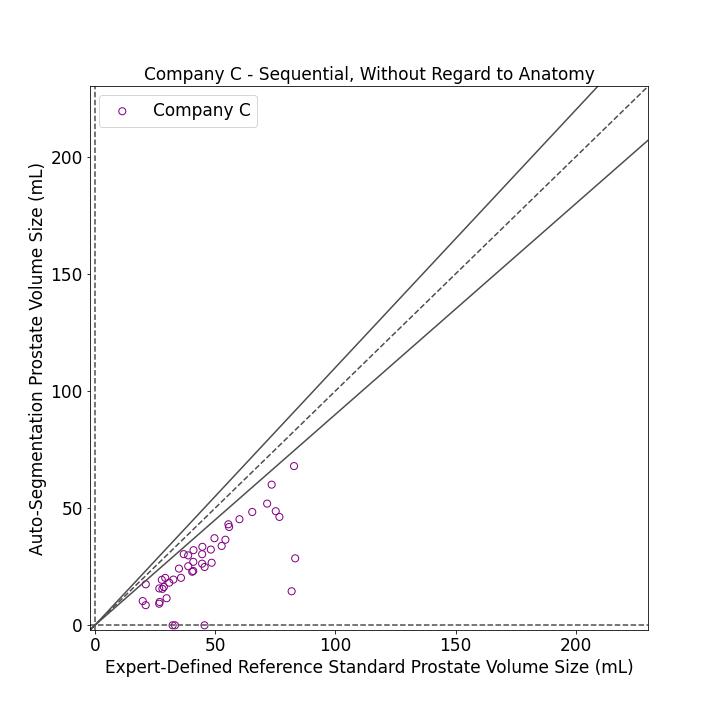**  F  Company C |
| **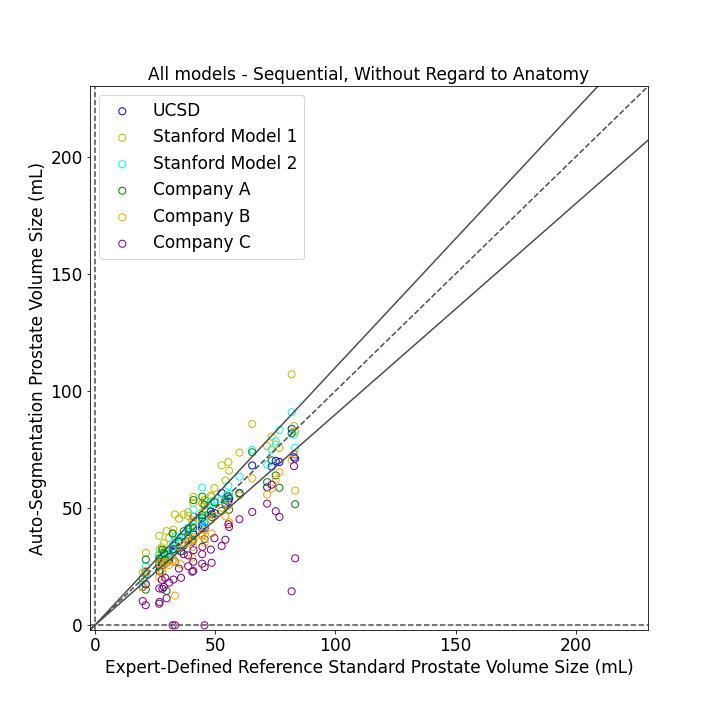**  G  All Models | |

Figure E2

Scatter plot of auto-segmentation volume vs. expert-defined consensus contour for cases chosen without consideration of prostate anatomy. N=45 cases.

For all panels, the X-axis shows the absolute prostate volume (mL) of the expert-defined consensus contour while the Y-axis shows the absolute prostate volume (mL) of the auto-segmentation product for the cases selected without consideration of prostate anatomy. Panel G compares all auto-segmentation models versus the consensus contour while panels A-F compare each model individually against the expert contour. Blue: UCSD. Yellow: Stanford Model 1. Cyan: Stanford Model 2. Green: Company A’s product. Orange: Company B’s product. Purple: Company C’s product.

| **Scanner** | **UCSD*** | **MGH** | **URMC** | **UCSF** | **UTHSCSA** | **Cambridge** |
| --- | --- | --- | --- | --- | --- | --- |
| **Pulse sequence** | Fast Spin Echo (FSE) | Turbo Spin Echo (FSE) | Fast Spin Echo (FSE) | Fast Spin Echo (FSE) | Fast Spin Echo (FSE) | Turbo Spin Echo (FSE) |
| **TR (ms)** | 5300 | 4800 | 4800 | 2964 | 3130 | 4710 |
| **TE (ms)** | 100 | 104 | 104 | 150 | 98 | 100 |
| **FOV (mm)** | 200 x 200 | 180 x 180 | 180 x 180 | 220 x 220 | 180 x 180 | 180 x 180 |
| **Matrix [resampled dimensions]** | 320 x 320 [512 x 512] | 384 x 365 [384 x 384] | 384 x 365 [384 x 384] | 512 x 512 [320 x 320] | 448 x 256 [512 x 512] | 240 x 320 [320 x 320] |
| **Slices** | 32 | 32 | 32 | 36 | 30 | 30 |
| **Slice Thickness (mm）** | 3 | 3 | 3 | 3 | 3 | 3 |
| **Field Strength (T)** | 3 | 3 | 3 | 3 | 3 | 3 |

Table E2. MRI acquisition parameters *T_2_*-weighted sequences for each cohort. TR: repetition time. TE: echo time. FOV: field-of-view. FSE: fast spin echo. *One of the ten patients from UCSD was scanned with a TR of 7000 ms and FOV 240 x 240 mm

| **Model** | **Dice** | | | **Max error outside prostate (mm)** | | | **Max error inside prostate (mm)** | | | **Average error (mm)** | | |
| --- | --- | --- | --- | --- | --- | --- | --- | --- | --- | --- | --- | --- |
| **Model** | median | | | median | | | median | | | median | | |
|  | min | IQR | max | min | IQR | max | min | IQR | max | min | IQR | max |
| **UCSD** | 0.95 | | | 3.2 | | | 4.4 | | | 1.3 | | |
|  | 0.87 | 0.94-0.96 | 0.97 | 1.9 | 3.0-5.0 | 6.8 | 0.5 | 3.5-6.0 | 10.3 | 1.0 | 1.1-1.4 | 2.4 |
| **Stanford Model 1** | 0.90 | | | 7.3 | | | 3.7 | | | 2.0 | | |
|  | 0.75 | 0.88-0.92 | 0.94 | 4.2 | 6.0-8.5 | 15.5 | 1.5 | 3.0-9.0 | 16.7 | 1.4 | 1.8-2.3 | 4.6 |
| **Stanford Model 2** | 0.93 | | | 9.0 | | | 3.9 | | | 2.0 | | |
|  | 0.76 | 0.92-0.94 | 0.95 | 6.0 | 6.7-15.0 | 19.4 | 1.6 | 3.0-4.8 | 11.0 | 1.4 | 1.6-2.4 | 3.7 |
| **Company A** | 0.90 | | | 4.4 | | | 6.0 | | | 1.7 | | |
|  | 0.76 | 0.87-0.91 | 0.94 | 2.9 | 3.3-6.0 | 15.8 | 2.8 | 4.3-6.6 | 10.7 | 1.3 | 1.5-2.0 | 3.3 |
| **Company B** | 0.90 | | | 6.1 | | | 7.8 | | | 1.9 | | |
|  | 0.79 | 0.89-0.92 | 0.93 | 3.1 | 5.0-8.5 | 12.8 | 4.0 | 6.0-9.0 | 11.2 | 1.5 | 1.7-2.2 | 3.1 |
| **Company C** | 0.85 | | | 3.2 | | | 0.7 | | | 2.2 | | |
|  | 0.41 | 0.82-0.87 | 0.91 | 0.0 | 2.8-5.2 | 17.8 | 6.0 | 7.0-9.6 | 24.2 | 1.7 | 2.2-3.0 | 7.1 |
| **Model** | **Dice-main** | | | **Difference in superior extent of Contour (slice)** | | | **Difference in inferior extent of contour (slice)** | | | **Volume difference (%)** | | |
| **Model** | median | | | median | | | median | | | median | | |
|  | min | IQR | max | min | IQR | max | min | IQR | max | min | IQR | max |
| **UCSD** | 0.95 | | | 0 | | | 1 | | | 3.3 | | |
|  | 0.90 | 0.95-0.96 | 0.98 | 0 | 0-1 | 2 | 0 | 0-1 | 2 | 0.1 | 2.1-7.7 | 27.1 |
| **Stanford Model 1** | 0.92 | | | 1 | | | 1 | | | 12.5 | | |
|  | 0.81 | 0.91-0.93 | 0.95 | 0 | 0-1 | 3 | 0 | 1-2 | 5 | 5.1 | 9.8-19.2 | 64.7 |
| **Stanford Model 2** | 0.95 | | | 1 | | | 3 | | | 5.3 | | |
|  | 0.79 | 0.94-0.96 | 0.96 | 0 | 0-1 | 4 | 1 | 2-4 | 6 | 0.1 | 3.0-7.4 | 36.1 |
| **Company A** | 0.91 | | | 0 | | | 1 | | | 13.0 | | |
|  | 0.81 | 0.89-0.93 | 0.95 | 0 | 0-1 | 3 | 0 | 0-1 | 3 | 0.6 | 5.6-19.8 | 54.5 |
| **Company B** | 0.92 | | | 1 | | | 1 | | | 10.6 | | |
|  | 0.80 | 0.91-0.93 | 0.93 | 0 | 1-2 | 4 | 0 | 0-1 | 3 | 2.2 | 8.8-14.4 | 31.0 |
| **Company C** | 0.88 | | | 1 | | | 2 | | | 21.6 | | |
|  | 0.41 | 0.86-0.89 | 0.93 | 0 | 1-2 | 3 | 1 | 2-3 | 5 | 1.6 | 17.8-27.5 | 75.0 |

Table E3. Accuracy metrics for only cases with a prominent median lobe of the prostate (N=23). Difference in superior extent of contour and difference in inferior extent of contour are measured in number of slices (slices are 3 mm thick). Volume difference (%) of each model is how much the model’s estimate of the prostate volume differed from the reference standard volume. The median, min, IQR, and max refer to across patients for that metric/model combination.

| **Model** | **Dice** | | | **Max error outside prostate (mm)** | | | **Max error inside prostate (mm)** | | | **Average error (mm)** | | |
| --- | --- | --- | --- | --- | --- | --- | --- | --- | --- | --- | --- | --- |
| **Model** | median | | | median | | | median | | | median | | |
|  | min | IQR | max | min | IQR | max | min | IQR | max | min | IQR | max |
| **UCSD** | 0.93 | | | 3.0 | | | 3.7 | | | 1.3 | | |
|  | 0.89 | 0.92-0.95 | 0.96 | 1.3 | 3.0-3.8 | 7.8 | 1.6 | 3.0-6.0 | 7.1 | 0.9 | 1.2-1.5 | 2.1 |
| **Stanford Model 1** | 0.88 | | | 6.6 | | | 3.0 | | | 1.9 | | |
|  | 0.81 | 0.96-0.90 | 0.94 | 3.2 | 6.0-9.0 | 15.7 | 0.6 | 2.4-3.6 | 10.4 | 1.2 | 1.7-2.2 | 3.8 |
| **Stanford Model 2** | 0.91 | | | 9.4 | | | 3.2 | | | 2.1 | | |
|  | 0.64 | 0.90-0.93 | 0.95 | 3.0 | 6.7-12.5 | 30.0 | 1.9 | 3.0-4.7 | 8.5 | 1.1 | 1.6-2.6 | 8.3 |
| **Company A** | 0.89 | | | 4.3 | | | 4.3 | | | 1.6 | | |
|  | 0.65 | 0.87-0.91 | 0.94 | 1.7 | 3.2-5.7 | 9.6 | 1.6 | 3.2-5.2 | 11.0 | 1.1 | 1.4-2.0 | 3.0 |
| **Company B** | 0.87 | | | 4.5 | | | 6.2 | | | 1.9 | | |
|  | 0.34 | 0.85-0.90 | 0.93 | 2.7 | 3.6-6.0 | 21.3 | 3.7 | 5.8-8.1 | 14.9 | 1.3 | 1.8-2.2 | 3.9 |
| **Company C** | 0.75 | | | 3.0 | | | 8.5 | | | 2.4 | | |
|  | 0.00 | 0.71-0.82 | 0.87 | 0.0 | 1.5-3.6 | 41.4 | 0.0 | 6.8-9.3 | 14.7 | 0.0 | 2.1-2.8 | 30.7 |
| **Model** | **Dice-main** | | | **Difference in superior extent of contour (slices)** | | | **Difference in inferior extent of contour (slices)** | | | **Volume difference (%)** | | |
| **Model** | median | | | median | | | median | | | median | | |
|  | min | IQR | max | min | IQR | max | min | IQR | max | min | IQR | max |
| **UCSD** | 0.95 | | | 1 | | | 1 | | | 4.8 | | |
|  | 0.92 | 0.94-0.96 | 0.97 | 0 | 0-1 | 2 | 0 | 1-2 | 3 | 0.1 | 3.3-9.3 | 17.8 |
| **Stanford Model 1** | 0.92 | | | 0 | | | 1 | | | 17.7 | | |
|  | 0.86 | 0.90-0.93 | 0.96 | 0 | 0-1 | 2 | 0 | 1-2 | 4 | 4.9 | 10.6-25.5 | 45.9 |
| **Stanford Model 2** | 0.94 | | | 1 | | | 3 | | | 4.2 | | |
|  | 0.72 | 0.93-0.95 | 0.96 | 0 | 0-1 | 4 | 0 | 2-4 | 9 | 0.5 | 2.0-8.1 | 31.8 |
| **Company A** | 0.92 | | | 1 | | | 1 | | | 7.4 | | |
|  | 0.67 | 0.90-0.93 | 0.95 | 0 | 0-1 | 2 | 0 | 0-1 | 3 | 0.1 | 4.2-14.9 | 50.7 |
| **Company B** | 0.90 | | | 1 | | | 1 | | | 13.8 | | |
|  | 0.36 | 0.88-0.92 | 0.95 | 0 | 1-2 | 8 | 0 | 0-1 | 5 | 0.8 | 7.7-18.7 | 62.0 |
| **Company C** | 0.78 | | | 1 | | | 3 | | | 35.8 | | |
|  | 0.00 | 0.74-0.86 | 0.89 | 0 | 0-2 | 4 | 0 | 2-4 | 9 | 17.5 | 26.1-45.0 | 100.0 |

Table E4. Accuracy metrics for only cases selected at random, without regard to anatomy (N=45). Difference in superior extent of contour and difference in inferior extent of contour are measured in number of slices (slices are 3 mm thick). Volume difference (%) of each model is how much the model’s estimate of the prostate volume differed from the reference standard volume. The median, min, IQR, and max refer to across patients for that metric/model combination.

| **Institution** | **T3a Carcinomas cases confirmed** |
| --- | --- |
| **UCSD** | 0 |
| **MGH** | 1 |
| **URMC** | Clinical Information Not Available |
| **UCSF** | 4 |
| **UTHSCSA** | Clinical Information Not Available |
| **Cambridge** | Clinical Information Not Available |
| **Total** | **5** |

Table E5. T3a Carcinomas cases. Clinical information is not available for the 34 cases from URMC, UTHSCSA, and Cambridge. There are no T3a carcinomas in the 14 cases UCSD. Out of 10 cases from MGH, there is 1 confirmed T3a carcinoma. Of the 10 cases from UCSF, there are 4 confirmed cases of T3a carcinoma.

| **Model** | **Dice** | | | **Max error outside prostate (mm)** | | | **Max error inside prostate (mm)** | | | **Average error (mm)** | | |
| --- | --- | --- | --- | --- | --- | --- | --- | --- | --- | --- | --- | --- |
| **Model** | median | | | median | | | median | | | median | | |
|  | min | IQR | max | min | IQR | max | min | IQR | max | min | IQR | max |
| **UCSD** | 0.94 | | | 6.0 | | | 6.0 | | | 1.6 | | |
|  | 0.90 | 0.93-0.94 | 0.95 | 3.0 | 3.0-6.1 | 7.8 | 3.0 | 3.3-6.0 | 7.1 | 1.3 | 1.4-1.7 | 1.7 |
| **Stanford Model 1** | 0.90 | | | 7.0 | | | 3.0 | | | 2.0 | | |
|  | 0.86 | 0.89-0.91 | 0.94 | 6.0 | 6.9-7.9 | 11.0 | 3.0 | 3.0-8.1 | 10.2 | 1.7 | 1.9-2.3 | 2.4 |
| **Stanford Model 2** | 0.92 | | | 9.2 | | | 4.1 | | | 1.9 | | |
|  | 0.90 | 0.91-0.92 | 0.95 | 6.0 | 6.7-9.6 | 15.0 | 3.0 | 3.9-4.1 | 4.5 | 1.5 | 1.9-2.1 | 2.6 |
| **Company A** | 0.86 | | | 3.8 | | | 6.0 | | | 2.4 | | |
|  | 0.76 | 0.84-0.89 | 0.90 | 1.7 | 3.0-4.4 | 9.6 | 3.0 | 5.2-10.7 | 11.0 | 1.6 | 1.7-2.5 | 2.7 |
| **Company B** | 0.86 | | | 5.5 | | | 8.3 | | | 2.3 | | |
|  | 0.81 | 0.85-0.87 | 0.89 | 3.7 | 5.0-6.0 | 6.8 | 6.2 | 6.6-9.3 | 10.8 | 1.9 | 2.2-2.5 | 2.7 |
| **Company C** | 0.71 | | | 2.3 | | | 11.2 | | | 3.1 | | |
|  | 0.41 | 0.51-0.72 | 0.81 | 0.0 | 0.0-3.0 | 5.2 | 8.1 | 9.0-14.7 | 24.2 | 2.3 | 2.7-4.6 | 7.1 |
| **Model** | **Dice-main** | | | **Difference in superior extent of Contour (slice)** | | | **Difference in inferior extent of contour (slice)** | | | **Volume difference (%)** | | |
| **Model** | median | | | median | | | median | | | median | | |
|  | min | IQR | max | min | IQR | max | min | IQR | max | min | IQR | max |
| **UCSD** | 0.95 | | | 1 | | | 2 | | | 4.7 | | |
|  | 0.92 | 0.95-0.96 | 0.96 | 0 | 1-1 | 1 | 0 | 1-2 | 2 | 0.2 | 2.4-4.7 | 14.6 |
| **Stanford Model 1** | 0.92 | | | 0 | | | 2 | | | 12.9 | | |
|  | 0.90 | 0.92-0.94 | 0.95 | 0 | 0-0 | 1 | 0 | 2-2 | 2 | 0.5 | 9.2-16.4 | 27.4 |
| **Stanford Model 2** | 0.94 | | | 1 | | | 3 | | | 4.3 | | |
|  | 0.93 | 0.93-0.95 | 0.95 | 0 | 0-1 | 2 | 2 | 2-3 | 5 | 1.4 | 2.0-6.8 | 9.1 |
| **Company A** | 0.87 | | | 1 | | | 1 | | | 22.8 | | |
|  | 0.78 | 0.85-0.91 | 0.93 | 0 | 0-1 | 2 | 0 | 0-1 | 2 | 1.5 | 4.8-23.4 | 38.0 |
| **Company B** | 0.89 | | | 2 | | | 1 | | | 18.2 | | |
|  | 0.82 | 0.88-0.90 | 0.91 | 0 | 2-2 | 3 | 0 | 1-2 | 3 | 14.6 | 14.6-19.2 | 31.0 |
| **Company C** | 0.75 | | | 1 | | | 2 | | | 45.0 | | |
|  | 0.41 | 0.52-0.75 | 0.85 | 1 | 1-3 | 4 | 2 | 2-5 | 5 | 25.1 | 43.3-65.7 | 74.6 |

Table E6. Accuracy metrics for the cases with T3a Carcinomas. Difference in superior extent of contour and difference in inferior extent of contour are measured in number of slices (slices are 3 mm thick). Volume difference (%) of each model is how much the model’s estimate of the prostate volume differed from the reference standard volume.
